# SVD-based filtering to detect intraplaque hemorrhage using single wavelength photoacoustic imaging

**DOI:** 10.1117/1.JBO.26.11.116003

**Published:** 2021-11-06

**Authors:** Roy van Hees, Jan-Willem Muller, Frans van de Vosse, Marcel Rutten, Marc van Sambeek, Min Wu, Richard Lopata

**Affiliations:** a Eindhoven University of Technology, Photoacoustics and Ultrasound Laboratory Eindhoven (PULS/e), Department of Biomedical Engineering, Eindhoven, The Netherlands; b Catharina Hospital, Eindhoven, The Netherlands

**Keywords:** singular value decomposition, intraplaque hemorrhage, photoacoustic imaging, single wavelength

## Abstract

**Significance:** Intraplaque hemorrhage (IPH) is an important indicator of plaque vulnerability. Early detection could aid the prevention of stroke.

**Aim:** We aim to detect IPH with single wavelength PA imaging *in vivo* and to improve image quality.

**Approach:** We developed a singular value decomposition (SVD)-based filter to detect the nonstationary and stationary components in ultrasound data. A PA mask was created to detect stationary (IPH) sources. The method was tested *ex vivo* using phantoms and *in vivo* in patients.

**Results:** The flow and IPH channels were successfully separated in the phantom data. We can also detect the PA signals from IPH and reject signals from the carotid lumen *in vivo*. Generalized contrast-to-noise ratio improved in both *ex vivo* and *in vivo* in US imaging.

**Conclusions:** SVD-based filtering can successfully detect IPH using a single laser wavelength, opening up opportunities for more economical and cost-effective laser sources.

## 1 Introduction

Stroke is the second leading cause of death and the third leading cause of disability worldwide.^1^ The major cause of stroke is the rupture of plaque in the carotid artery. These rupture-prone plaques (vulnerable plaques) are characterized by the presence of a lipid-rich core, a thin fibrous cap, and intraplaque hemorrhages (IPH).^2^ The current clinical guideline to determine plaque vulnerability, however, is based on the grade of stenosis, which is often assessed by duplex ultrasound (US).^3^ When the grade of stenosis is over 70% and less than 100%, the patient will undergo surgery.

The current diagnosis of vulnerable plaques based on ultrasound (US) characterized luminal stenosis severity leads to severe overtreatment. It is reported that only 16% of the endarterectomies performed based on clinical guidelines contained a vulnerable plaque.^4^ Current studies suggest that plaque vulnerability is related to its structure, geometry, and composition.^2^ The presence of neovascularization and IPHs is considered to be one of the key indicators for plaque vulnerability.^5^ Studies have found a correlation between the presence of IPH and heterogeneous US echo patterns,^6^^,^^7^ but other studies argue that IPH cannot be identified this way.^8^^,^^9^ Contrast-enhanced US has also been investigated^10^^,^^11^ but has not convincingly demonstrated the capability to detect IPH.^9^

By adding optical absorption contrast, photoacoustic (PA) imaging (PAI) can provide additional morphological information, to distinguish between tissue types. In contrast to US, which transmits an US pulse, PAI transmits a pulsed laser beam. The laser pulse propagates through the tissue and is absorbed by chromophores such as melanin or hemoglobin. This causes a local temperature increase which results in thermoelastic expansion and a subsequent transient pressure rise that will propagate through the tissue and can be recorded using a conventional US transducer.

The advantage of PAI compared with other purely optical techniques such as optical coherence tomography (OCT) is its relatively high penetration depth of several centimeters,^12^^,^^13^ as opposed to the penetration depth of about 2 mm for OCT.^14^ The last decade of PAI has seen a lot of research and development, with examples including brain and whole body imaging of small animals, dual-modal US/PA imaging in the clinic, improvements in frame rate and resolution of PA microscopy, the development of PA mesoscopy, advances in PA endoscopy (e.g., intravascular US/PA to detect lipids^15^^,^^16^), and the advent of wearable PA.^17^

Previous studies have shown that PAI is very sensitive to hemoglobin and can thereby be used to detect IPH.^18^^–^^20^ To be able to identify IPH *in vivo*, we must differentiate the PA signal generated by IPHs from the blood flow in the adjacent carotid lumen and/or other blood vessels such as arterioles and veins. This generally requires multiple laser wavelengths to either measure the blood oxygenation level^21^ or characterize the different hemoglobin types, such as deoxyhemoglobin and met-hemoglobin,^22^^–^^26^ which increases cost and complexity, and reduces PA imaging speed. Pulsed laser diodes can alleviate these issues and provide imaging frame rates of up to 7000 frames per second.^27^

A recent study by Das et al.^28^ has demonstrated a method for the detection of blood clots circulating at high speed using a single laser wavelength, but this method is better suited to diagnosing (recurring) deep vein thrombosis, in which clots have already entered the blood stream, whereas this study aims to detect IPH before it enters the blood stream. Another study by the same author^29^ demonstrated a method to determine whether a clot is rich in red blood cells and to estimate the approximate age of the clot. However, this was only demonstrated *in vitro* and is better suited to monitor treatments that employ clot-dissolving drugs.

In this study, we present a framework to detect IPH in an *in vivo* setting with a single wavelength. The framework is based on the detection of PA signals from hemoglobin, and the separation of hemorrhages from arterial and venous blood, based on singular value decomposition of the available, interleaved US data. To this end, a blockwise SVD method^30^ has been adapted for interleaved US/PA scans acquired with a low pulse repetition frequency (PRF) using a handheld US/PA probe. The method has been validated using phantom data and *in vivo* patient data. In general, this framework can improve image quality in both *in vivo* US and PA data and separate PA signals originating from stationary and nonstationary sources.

## 2 Material and Methods

### 2.1 Imaging Set-Up and Data Acquisition

A real-time PA imaging system with a handheld dual-mode US/PA probe was used for data acquisition. The handheld probe in the project is integrated with diode lasers (LUMIBIRD, France, $E_{\text{pulse},760\;\mathrm{nm}}=0.58\;\mathrm{mJ}$, $t_{\text{pulse}}\approx 60\;\mathrm{ns}$ FWHM, $\text{fluence}=0.81\;\mathrm{mJ}/{\mathrm{cm}}^{2}$, 1 kHz repetition rate), and a linear array US transducer (64 elements, $F_{c}=7.5\;\mathrm{MHz}$, 70% bandwidth, 245  μm pitch). The PA beam is angled 51 deg and intersects the US plane at a depth of ∼5  mm.^31^ The probe was connected to a MyLab One US scanner (Esaote, Genova, Italy, 12 bit digitization, maximum sampling frequency of 50 MHz) and controlled using a PC.

Data were gathered using interleaved plane wave US and PA acquisitions. The sampling frequency was above 22.2 MHz, and the frame rate varied between 10 and 30 frames per second.

### 2.2 Phantom Preparation and *Ex Vivo* Experiments

To validate the framework, we performed *ex vivo* experiments on cylindrical hemorrhage phantoms, designed to mimic a stenosed carotid artery that includes blood flow and hemorrhages (stationary). The phantoms were made using ∼15 weight-percent poly-vinyl-alcohol (Mowiol 28-99, Sigma-Aldrich, Zwijndrecht, the Netherlands) and 1.2 weight-percent Orgasol (ELF Atochem, Paris, France). These were mixed at 85°C with demi-water, then poured into a mold, and subjected to four freeze/thaw cycles, with at least 16 h of freezing each cycle.

**Fig. 1:**
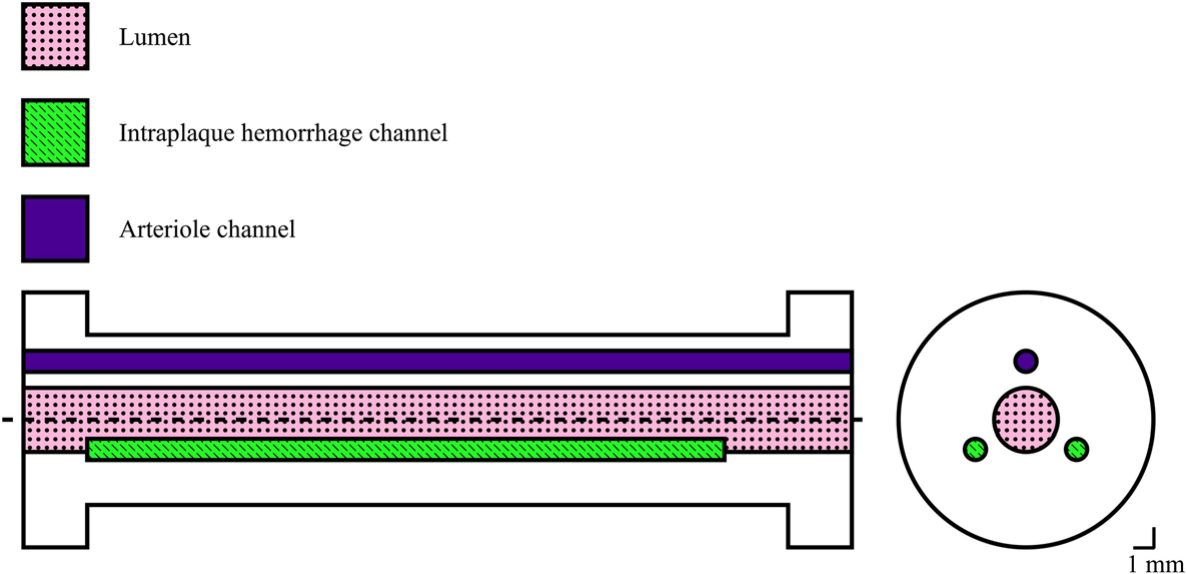
Phantom design schematic: vessel phantom with two IPH channels and one arteriole channel. The total length of the phantom is 39 mm and the diameter is 8 mm.

Figure 1 shows an overview of the design. The phantoms are 39 mm long, have a 3-mm central lumen, and three cylindrical cavities evenly distributed around the lumen. The central lumen was perfused using pulsatile flow to mimic the common carotid’s lumen, which will act as a nonstationary source of PAI signals generated by the hemoglobin. Two of the cavities with a fixed diameter of 1 mm and a length of ∼17  mm contained sealed aged blood to mimic IPHs (i.e., stationary PA sources). The third cylindrical cavity had a diameter varying from 0.25 to 1.00 mm, in 0.25 mm increments, one for each of the four different phantoms and was perfused with a constant flow to mimic small vessels such as an arteriole (i.e., a nonstationary PA source).

**Fig. 2:**
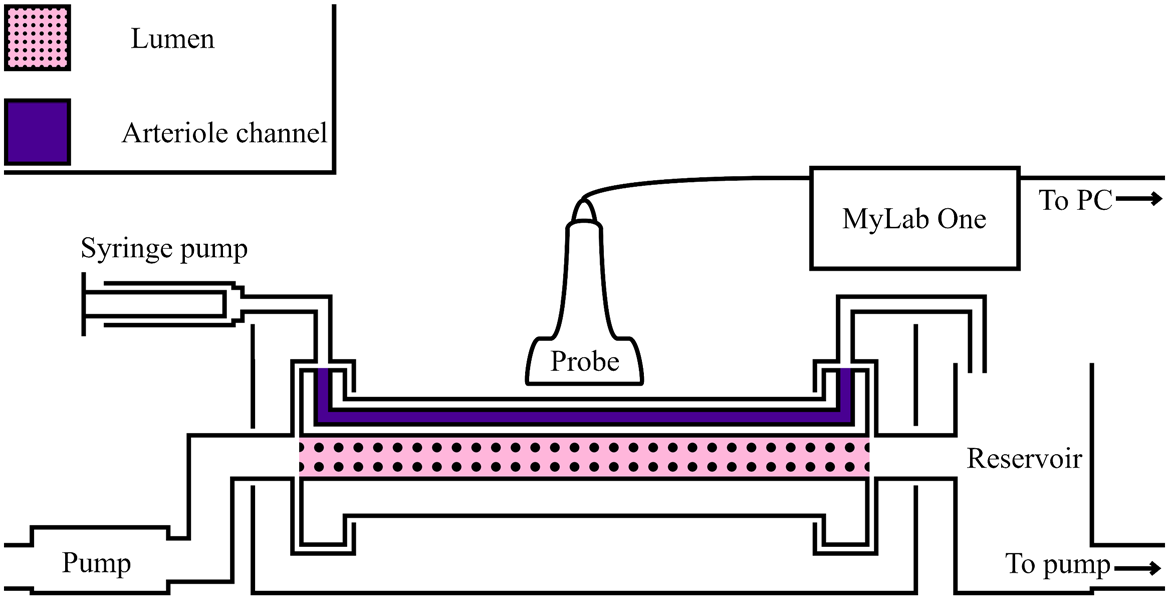
Schematic overview of the mock loop set-up.

The phantom was placed in a mock loop set-up in a water tank at room temperature, and anticoagulated porcine blood was pumped through the lumen and arteriole channel, as shown in Fig. 2. The lumen channel was perfused using a peristaltic water pump operating at a frequency of 1 Hz, and the pressure level applied results in a circumferential inner wall strain of ∼9%. This is similar to the wall motion expected from a healthy volunteer, which is typically 7% to 13%.^32^ Please note that in this case, we tested the framework in more challenging conditions, as patients often have calcified blood vessels and thus normally show less wall motion, typically 0% to 3%.^32^

The arteriole channel was perfused using a syringe pump (Terufusion STC-521, Terumo Benelux, Leuven, Belgium) with a constant flow. To investigate the influence of size of the arteriole channel, we tested the four phantoms, all with the same flow of 10  mL/h. Therefore, except for the phantom with an arteriole size of 0.25 mm, the flow velocity is below the peak flow velocity of an arteriole (typically 0.5 to 1  cm/s). This makes the conditions for the proposed framework more challenging for the 1.00, 0.75, and 0.50 mm arteriole channel sizes, because the flow velocity is closer to the wall motion speed of the IPH channel. It is less challenging for the 0.25-mm arteriole channel size.

**Fig. 3:**
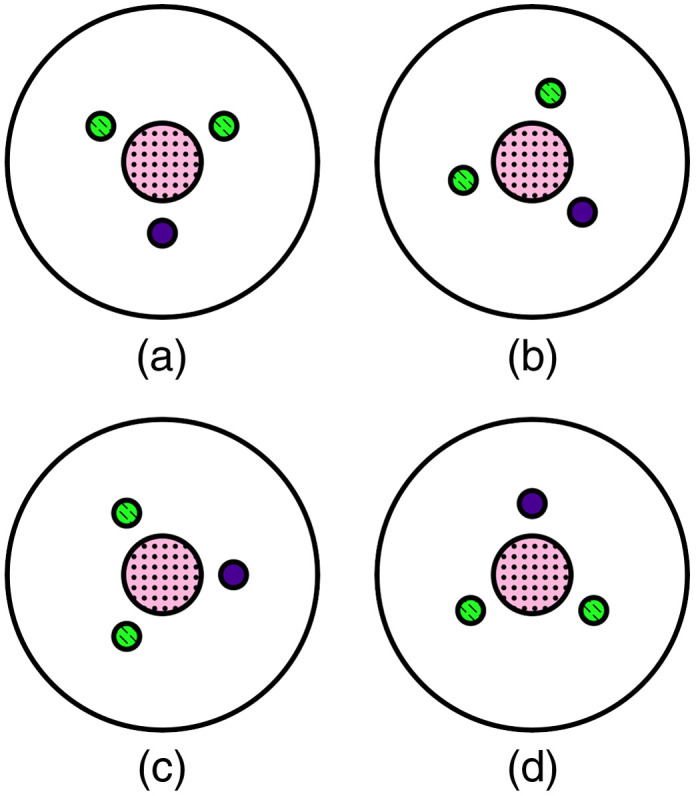
Arteriole channel orientations: (a) channel on the bottom, (b) channel on the bottom right, (c) channel on the right, and (d) channel on top.

In every experiment, a total of 4000 acquisitions were recorded, corresponding to at least 300 compounded US and PA images, that were used for further processing. In addition, the influence of spatial variation in fluence distribution was investigated for the 0.50-mm arteriole channel size by changing the orientation of the phantom as shown in Fig. 3.

### 2.3 *In Vivo* PA Imaging of Patients

*In vivo* US/PA imaging was performed in patients during a carotid endarterectomy surgery, a study in collaboration with the Catharina hospital in Eindhoven (CZE). The study was approved by the local Ethical Committee (MEC-U).

After obtaining informed consent from the patient, the patient underwent a normal endarterectomy procedure, after applying general anesthesia. A layer of gel was applied to both the US transducer and the PA optical window, after which the probe was sealed in a transparent, sterile probe sleeve. Before clamping of the carotid bifurcation, the cavity surrounding the bifurcation was filled with saline, the probe was placed in the cavity, and US/PA imaging was performed without any contact between the probe and the carotid artery, which showed both blood flow and normal *in vivo* wall motion. During imaging, annotations of the imaging location were made using the bifurcation as landmark. The imaging was performed by the acting surgeon, who is experienced in both US and PAI, supported by a small team of US/PA technicians. Each imaging segment was limited to a maximum of 800 US and PA frames at 30 Hz, to limit the impact on the endarterectomy procedure.

### 2.4 Blockwise SVD Algorithm

The idea to use singular value decomposition for clutter filtering in Doppler applications was originally introduced by Demené et al.,^33^ who proposed that an US signal can be described as a summation of clutter signal (later referred to as the stationary/tissue component in this paper), blood signal (later referred to as the flow component in this paper), and thermal/electrical noise. By removing the high (low-order) singular values, the clutter signal can be removed.

Later, Song et al.^30^ expanded on this concept. They proposed to apply the SVD in a blockwise manner, instead of to the whole image, because in US data, the signal-to-noise ratio is not constant but rather a function of depth. Furthermore, their paper introduced a method for automatically selecting thresholds for the singular values to separate all three signal components.

In this paper, we further expand on this concept and propose an SVD-based framework to robustly work with frame rates as low as 10 Hz, improve image quality, and separate PAI signals generated by nonstationary and stationary structures with a single wavelength, e.g., to detect IPHs.^18^ After the RF data acquisition, a conventional (delay-and-sum) beamformer was applied to construct the images. In the same acquisition sequence, the US acquisitions were coherently compounded into a single US frame, and the consecutive two PA single-wavelength acquisitions were averaged. Envelope detection and log compression were applied, after which the two-dimensional (2-D) multiframe data entered the SVD framework.

**Fig. 4:**
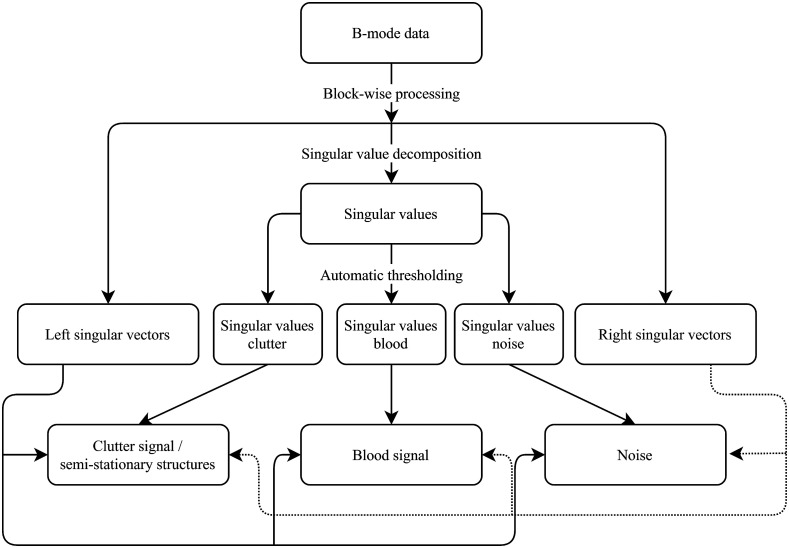
SVD data processing pipeline.

**Fig. 5:**
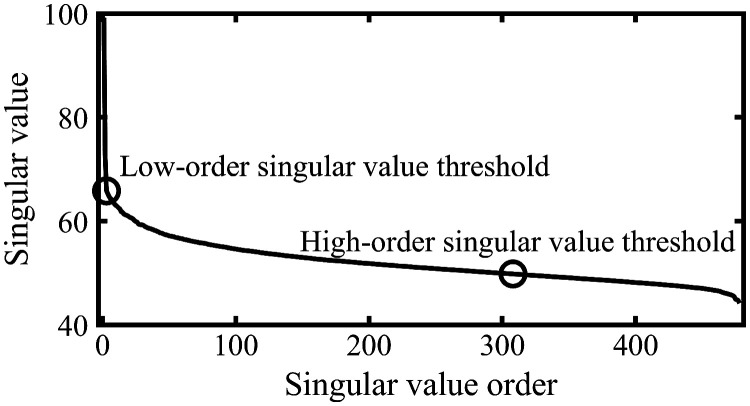
Example of a singular value curve and the selected thresholds. Singular values to the right of the second marker [located at (308, 50)] on the graph are categorized as noise. All points between the second marker and up to (but not including) the first marker [located at (3, 66)] are categorized as blood/perfusion signals, whereas all points to the left of (and including) the first marker are categorized as (semi-)stationary tissue signals.

Figure 4 shows a block diagram showing the data processing pipeline. After applying the singular value decomposition, the data can be divided into three components: highly spatial coherent US signals from (semi-)stationary tissues, US signals from the regions with perfusion, and thermal/electrical noise. Two (low- and high-order) singular value thresholds were used in the framework to separate between these components, as shown in Fig. 5.

The determination of the two thresholds is described in the following steps:

1. Singular value curves are calculated for all blocks, then averaged using a 2-D moving average filter, to smoothen the curves. The block-size and the moving average filter size are adjustable based on experimental conditions.
2. The low-order singular value threshold’s location is determined by the ratio between the current singular value and the next. It is placed at the point $S_{\mathrm{db}}(i)$, where the aforementioned ratio drops below a preset value for the first time:
  $$\frac{S_{\mathrm{db}}(i)}{S_{\mathrm{db}}(i+1)}<\text{preset}$$ (1)
  where i is the current singular value order and $S_{\mathrm{db}}(i)$ is the singular value in decibels for singular value order i. The preset value is highly dependent on US image quality.
3. To determine the location of the high-order singular value threshold, the singular value curve is first smoothened using a moving average filter with a length of five samples. Next, the first-order finite differences are calculated and smoothened with a moving average once more. The threshold is placed at point $S_{\mathrm{db}}(i)$, where the resulting curve has a global minimum. The recommended length of the moving average filter is 25% to 35% of the length of the singular value curve.
4. Once each block has a low-order and high-order singular value threshold, a median filter is moved over the blocks to remove outliers produced during the thresholding process, while retaining edges created by tissue boundaries. The size of the median filter is set to the dimensions of the moving average filter in step 1.

#### 2.4.1 Stationary PA source detection

**Fig. 6:**
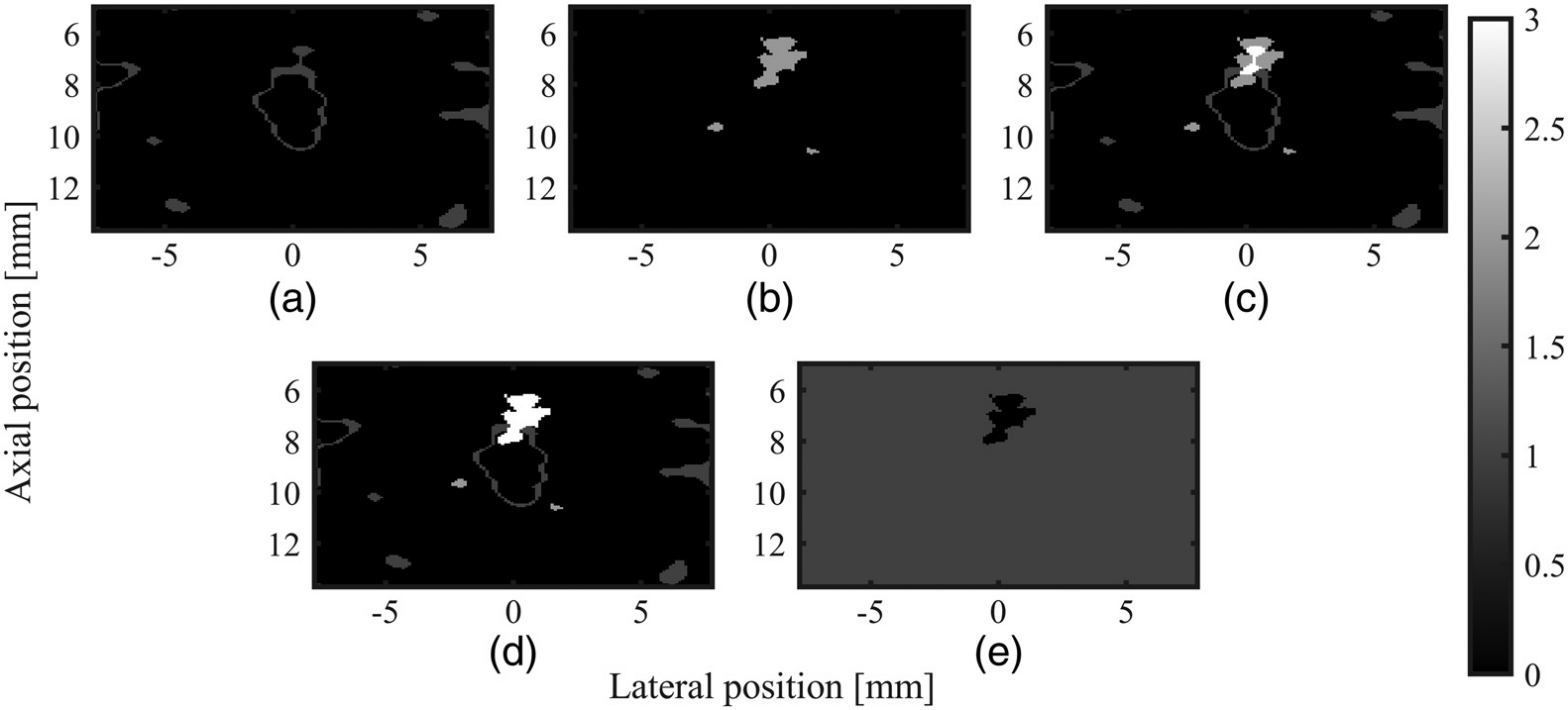
Mask creation overview. (a) The perfusion mask and (b) the thresholded PA signals are combined by means of addition to create (c). Next, (d) is created by iteratively growing the overlap area, by incrementing pixels that have a value of 2 and have a pixel with a value of 3 neighboring them. (e) The final mask is created by thresholding (d) and inverting the mask.

The defined thresholds are used to separate the US data into stationary, nonstationary (i.e., flow), and noise components. Based on the separation of these components, we can detect stationary PAI sources (i.e., intraplaque hemorrhages) using the following scheme:

1. A binary mask, zero in regions with perfusion, one otherwise, is created using the flow component of the US data: first, Gaussian blurring is applied to the US flow component, and then the temporal standard deviation is calculated and the dynamic range is compressed. Next, edge detection is applied. Small edges are removed using a standard image closing function.
2. Although the location of PA signals generally corresponds to the structures in the coregistered US data, there can be a mismatch between areas with significant PA signal and the corresponding US structures. To better mask the flow signals in the PA images, the overlap region needs to be adjusted. To this end, thresholding is applied to the PA data and overlaid on the binary US flow mask. Next, the region of overlap is enlarged until it completely matches the area of the PA signal. The process of mask creation is also shown in Fig. 6.
3. After filtering the flow signals in the PA data with the aforementioned mask, the hemorrhage signals are preserved and can be easily identified.

### 2.5 Framework Evaluation

As noted in the study by Demené et al.,^33^ clutter filtering for flow detection is a major challenge, especially for small vessels. We evaluated the capability of the SVD framework to distinguish between PAI signals of moving and stationary blood, with four phantoms that differed in arteriole channel size. One channel size was evaluated for different spatial fluence distributions, block-sizes, and execution time. The framework was also applied to *in vivo* patient PAI data to demonstrate its capabilities *in vivo*.

To quantify the improvement of the SVD framework on image quality, the generalized contrast to-noise ratio (gCNR) was examined.^34^ Compared with traditional methods, such as the signal-to-noise ratio or the contrast-to-noise ratio, the gCNR “… is resistant to dynamic range alterations … can be estimated on all kinds of images, regardless of compression, scale, or output units.” This makes it a better tool for assessment of imaging quality improvement with nonlinear filters, such as the proposed framework in this study.

The gCNR is defined as

gCNR=1−η, (2)

where η is the fractional overlap between the histograms of the signal and background and is defined as

$$\eta =\overset{N-1}{\underset{k=0}{\sum }}\min\{h_{\text{signal}}(x_{k}),h_{\text{background}}(x_{k})\},$$ (3)

where N is the number of bins, with the bins centered at $\left\{x_{0},x_{1},\ldots ,x_{N-1}\right\}$ and the associated histograms h in the respective region of interest. The gCNR therefore has a range between 0 and 1 and measures the fraction of pixels that are correctly identified by an ideal observer.

The application of different block-sizes can affect both image quality and PA signal separation. To investigate this, we evaluated filtering results for seven different block-sizes: 128 by 126 (entire field of view), 128 by 80, 128 by 40, 128 by 20, 64 by 80, 64 by 40, and 64 by 20 pixels.

The gCNR was evaluated in the 0.50-mm arteriole channel phantom, for both the stationary and flow component. For the flow component, we only evaluated the arteriole channel, because this is the most challenging area in flow detection, due to the much lower flow compared with the lumen channel. The arteriole channel gCNR was evaluated in the four different orientations, after taking the temporal standard deviation, but before dynamic range compression (discussed in Sec. 2.4.1). For the stationary US component, three random frames were selected, and for each frame the gCNR was evaluated in three locations, for a total of nine samples. An example is shown in Fig. 9(b).

## 3 Results

### 3.1 *Ex Vivo* Phantom Results

#### 3.1.1 Procedural results

Experiments were performed on each of the phantoms. Apart from the 0.25-mm channel phantom, all experiments yielded results that were fit for further processing. Due to the system’s spatial resolution being too low (i.e., 0.2 mm), the 0.25-mm channel phantom data could not be separated and were not processed further.

#### 3.1.2 Separation of PAI signals originating from stationary and nonstationary sources

**Fig. 7:**
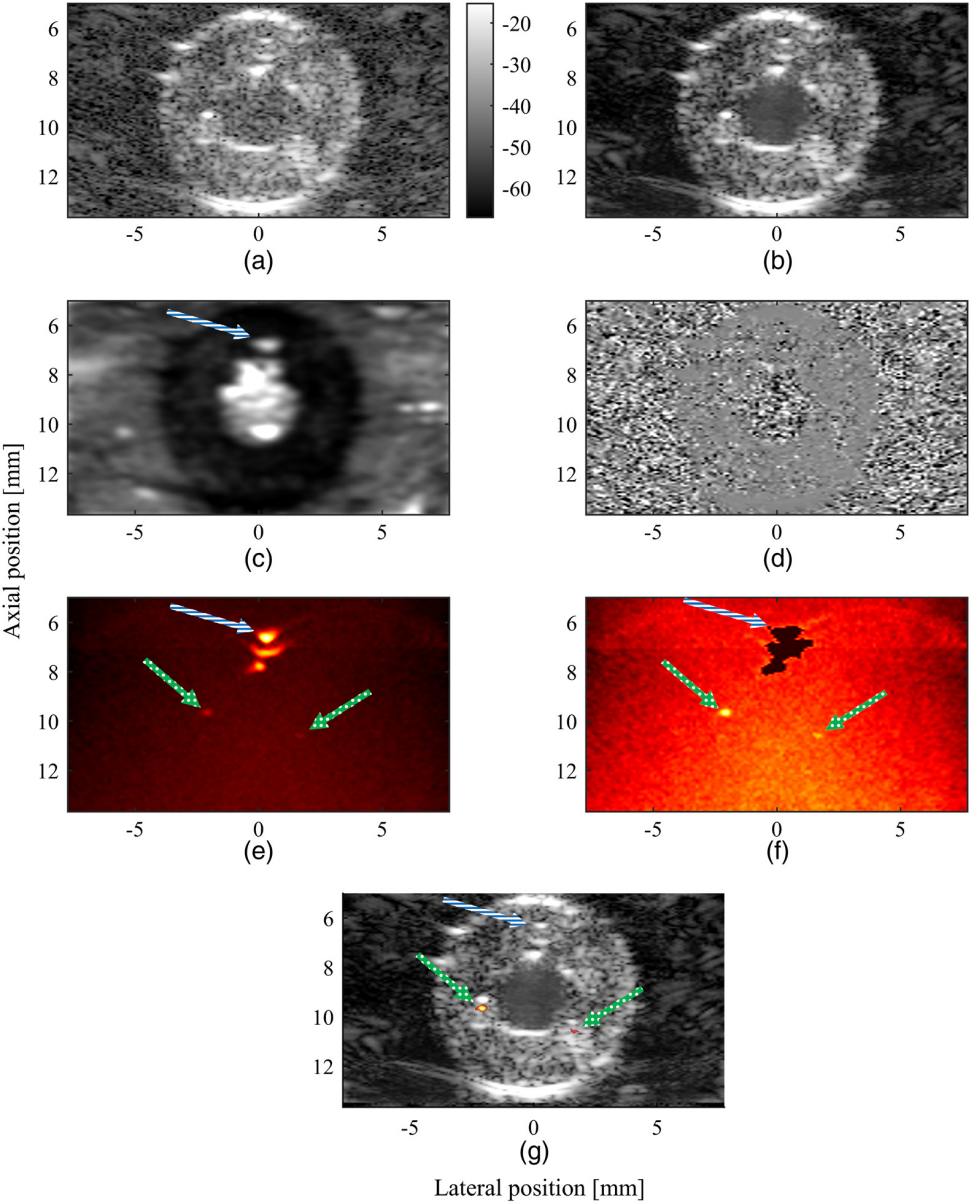
Separation of US and PA signals from stationary and nonstationary sources for the 0.75 mm channel size. (a) Original B-mode US data. The stationary, flow, and noise components after SVD filtering are shown in panels (b), (c), and (d), respectively. SVD noise filtered PA signals are shown in (e), whereas (f) shows the SVD noise-filtered PA signals with nonstationary PA signals sources masked. These data are overlaid on the stationary component of the filtered US data to create the image in (g). The arteriole channel is indicated using a blue/white striped (b/w) arrow and the IPH channels are indicated using green/white dotted (g/w) arrows.

Figure 7 shows the separation of US and PA signals from stationary and nonstationary sources in a phantom with a 0.75-mm arteriole channel. The blood/flow US component is shown in Fig. 7(c). In it, the signals related to flowing blood, coming from the lumen and arteriole channel, were clearly detected, whereas the signals from the stationary components [Fig. 7(b)], such as carotid wall tissue, were removed. The blue/white striped (b/w) arrow indicates the arteriole channel. Figure 7(a) shows the original US data, and Fig. 7(d) shows the residual noise component.

Figure 7(e) shows the unmasked, SVD noise-filtered PA data. It should be noted that the top two PA signals both originate from the interface of the arteriole channel, due to the limited bandwidth of the probe. No color bar has been included, as the SVD noise filtering is a nonlinear operation, which yields pixel values with arbitrary units that cannot be compared directly to the a decibel scale or each other. Figure 7(f) shows the PAI data after masking the flow regions, with the stationary hemorrhage signals clearly visible, as indicated by the green/white dotted (g/w) arrows, whereas the nonstationary PA signals have been completely removed, as indicated by the b/w arrow. The final overview of the overlaid US and PA data is shown in Fig. 7(g).

**Fig. 8:**
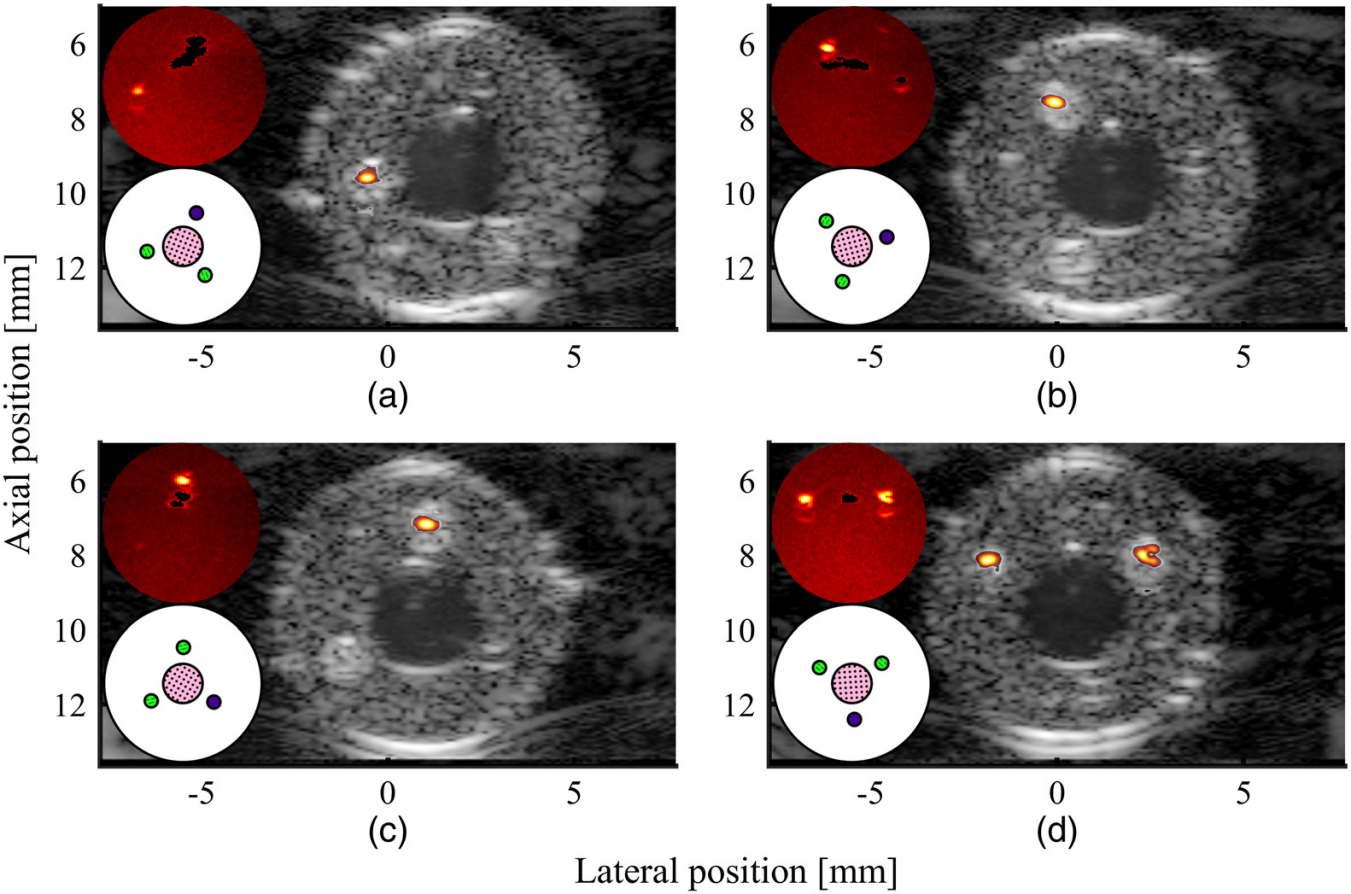
The influence of the spatial distribution of fluence on the flow detection algorithm of the SVD framework: arteriole channel (a) on the top (∼0  deg), (b) on the right side (∼90  deg), (c) on the bottom right (∼120  deg), and (d) on the bottom (∼180  deg). The top left overlays show the flow mask applied to the PA data to filter out the flow region. The bottom left overlays show the orientation of the phantom.

The framework can correctly filter out flow signals and detect IPHs with arteriole channel sizes of 1.00, 0.75 (Fig. 7), and 0.50 mm (Fig. 8).

#### 3.1.3 Influence of spatial variation in fluence distribution on intraplaque hemorrhage detection

Taking the 0.50-mm arteriole channel phantom as an example, the flow detection capability of the SVD framework for varying spatial distributions of fluence is shown in Fig. 8. PA signals are highly dependent on the local optical fluence: changing the orientation of the phantom allows to assess how the local illumination affects the IPH detection capability. As shown in Figs. 8(c) and 8(d), PA signals from channels located below the lumen cannot be detected.

As shown in the figure, as long as they are visible in the PA images, the flow channels (indicated by the small black mask shown at the top-left corner in each subfigure), and the hemorrhage channels, can be correctly differentiated. Thus, the nonstationary PAI signals, generated from the lumen and arteriole channel, were removed. PA signals from IPH ranging from a depth of 7.6 to 9.6 mm could be detected.

#### 3.1.4 Image quality improvement

**Fig. 9:**
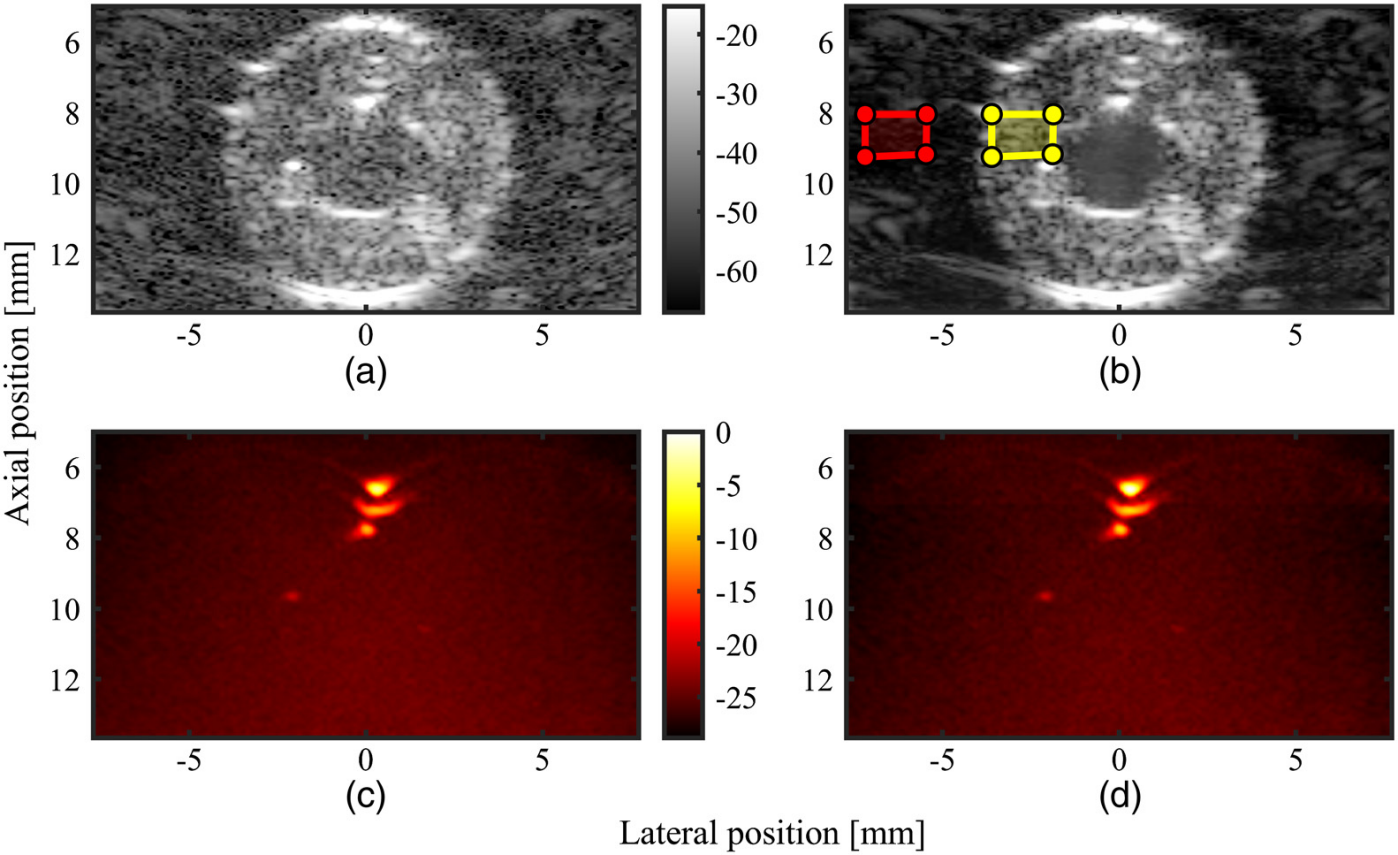
The 0.75-mm arteriole channel phantom. (a) Single frame B-mode US data (unfiltered), (b) single frame US stationary data, (c) 306 frames averaged non-SVD-filtered B-mode PA data (unfiltered), and (d) 306 frames averaged SVD noise-filtered PA stationary data. Contrast stretching has been applied to the US datasets, setting the dynamic range to the 1st and 99th percentile of the input. The red and yellow boxes in panel (b) show the background and signal region, respectively, for the gCNR calculation.

Figure 9 shows the US and PA data from the phantom with a 0.75-mm arteriole channel size as an example to demonstrate the improvement in US and PA image quality with the proposed framework. The dynamic range is set to the first and 99th percentile of the signal values for all figures to provide a fair comparison, and Fig. 9(b) shows an example of the region of interest for signal and background, shown with yellow and red boxes, respectively. In the figure, the improvement in US image quality is clearly appreciated, and the gCNR of the stationary component increased by 7.0% and 1.6% for the 0.75 and 0.50 mm arteriole channel sizes, respectively. The gCNR of the PA data could not be assessed. Since the image quality improvement of US was assessed, we included Figs. 9(c) and 9(d) for the sake of completeness and to allow readers to compare PA image quality, despite their minor differences.

#### 3.1.5 Influence of block-size on image quality and PA signal separation

Table 1 shows the influence of block-size on execution time and gCNR for the stationary, flow, and SVD noise-filtered data.

**Table 1:** Influence of block-size on execution time and gCNR for the stationary component, the blood/flow component and the SVD-noise filtered (stationary + flow) component.

Block-size		gCNR (μ±σ)			Execution time
(pixels)	(mm)	Stationary	Flow	Noise filtered	
128 by 126	15.4 by 8.7	0.97 ± 0.02	0.85 ± 0.15	0.94 ± 0.02	4 s
128 by 80	15.4 by 5.5	0.97 ± 0.02	0.81 ± 0.16	0.94 ± 0.02	78 s
128 by 40	15.4 by 2.7	0.97 ± 0.02	0.76 ± 0.29	0.94 ± 0.04	50 s
128 by 20	15.4 by 1.3	0.97 ± 0.02	0.79 ± 0.15	0.93 ± 0.04	23 s
64 by 80	7.7 by 5.5	0.97 ± 0.01	0.79 ± 0.21	0.93 ± 0.02	29 min
64 by 40	7.7 by 2.7	0.98 ± 0.02	0.79 ± 0.16	0.93 ± 0.04	20 min
64 by 20	7.7 by 1.3	0.98 ± 0.02	0.81 ± 0.04	0.91 ± 0.05	11 min

In the table, compared with the gCNR of the original unfiltered B-mode data, which is 0.96±0.02, the gCNR of the noise filtered data is lower for all block-sizes. The gCNR of the stationary component, however, does show a significant increase over the gCNR of the B-mode data for all block-sizes. Image quality can therefore still be improved by only displaying the stationary component, since in practice flow info is only displayed after internal processing for Doppler applications, then displayed as color overlay or separate from the B-mode data, or not at all for non-Doppler applications.

The most important image quality performance metric is the gCNR of the flow component, followed by the gCNR of the stationary and noise-filtered SVD data. The optimal block-size for the *ex vivo* data is 128 by 126 pixels, followed closely by a block-size of 128 by 80 pixels. Execution time is also greatly influenced by the block-size: setting the lateral size equal to the field-of-view greatly reduces execution time. Finally, system constraints need to be considered: larger block-sizes will require more system memory.

The execution was performed in 10 batches, with each batch executing the seven block-sizes in a random order. The execution time shown is the average of 10 batches and rounded to the nearest minute or second. The PC used was equipped with an AMD Ryzen 5 3600 (all-core overclock: 4.3 GHz, infinity fabric overclock: 1900 MHz), a MSI B450-A Pro Max motherboard and two 16 GB DIMMS of RAM, overclocked to 3800 MHz running 16/19/21/40 primary timings. The OS was Windows 10 version 2004, 64 bits, running MATLAB R2017b update 9.

### 3.2 *In Vivo* Imaging Results

**Fig. 10:**
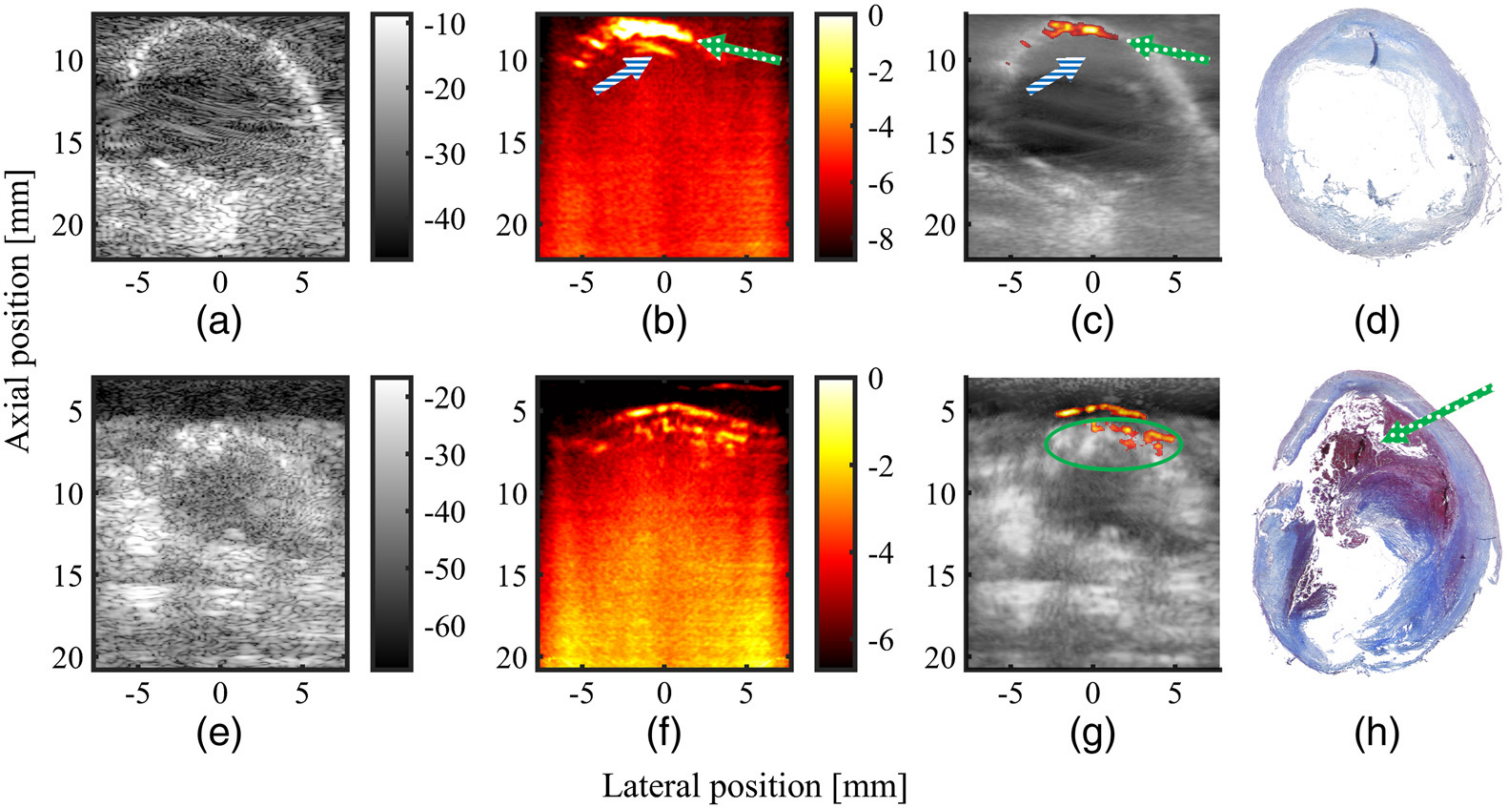
(a) Single frame B-mode US data, without SVD filtering; (b) 478 frames averaged B-mode PA data; (c) the SVD-filtered stationary component of the US data with the masked PA data overlaid; (d) histology image; (e) single frame B-mode US data; (f) 99 frames averaged B-mode PA data; (g) the stationary component of the US data with the masked PA data overlaid; and (h) histology image clearly showing the presence of IPH, as indicated by the g/w arrow. (a)–(d) Data from patient dataset 1; (e)–(h) data from patient dataset 2. Note how in panel (c), the PA data still show signals originating from the vessel wall (indicated by the g/w arrow), but not signals originating from the lumen (indicated by the b/w arrow), and how in panel (g) the IPH signal (indicated by the green circle) is still present.

Figures 10(a)–10(d) show the *in vivo* US and PA images of the common carotid artery obtained from patient dataset 1 (75 year old male). Histology, PA, and fused US/PA images of the common carotid obtained from patient dataset 2 (71 year old male) are shown in Figs. 10(e)–10(h). In patient dataset 1, there is no IPH in the image, and Fig. 10(c) shows that the PA signal originating from the lumen is correctly removed, whereas the PA signal originating from the vessel wall is retained. In patient dataset 2, a big IPH was clearly visible during the endarterectomy procedure of this particular patient. In the corresponding PA image, shown in Fig. 10(g), strong PA signals with a diffuse pattern were clearly observed, confirming the presence of IPH. These results demonstrate that the proposed method is capable of correctly identifying IPH in an *in vivo* setting.

It should also be noted that although a big IPH is present only in patient dataset 2, Figs. 10(c) and 10(g) show a comparable amount of PA signal. The strong PA signal from Fig. 10(c) is from the saline-artery wall interface, whereas the signal from Fig. 10(g) is from the plaque region and with a diffuse pattern, possibly due to the heterogeneous plaque composition. This observation is exactly in line with results from another recent study conducted by our group, which has a more comprehensive analysis of the *in vivo* PA signal from IPH.^35^

## 4 Discussion

In this study, we proposed a framework to separate PA signals originating from stationary and nonstationary PA sources, as well as increase image quality, and to improve *in vivo* US/PA imaging in general. PA signal separation was demonstrated for imaging of atherosclerotic plaques.

The proposed framework can successfully filter out blood flow and detect stationary PA sources such as IPHs for phantom datasets, even with arteriole channel diameters as small as 0.50 mm, and for different spatial distributions of fluence. It can increase gCNR by up to 7.0% for the *ex vivo* US data.

Moreover, the framework proposed can also be applied in *in-vivo* patient data despite challenging conditions, such as a limited PRF and acquisition time, thereby limiting the number of frames for processing, and motion of the probe due to handheld scanning in a space restricted cavity. It does not require accurate lumen segmentation to function, which is often difficult in practice and would require extra manual correction. US image quality was improved by 20% and the PA signals generated from carotid luminal flow were successfully removed. In the second *in vivo* patient dataset, a small part of the IPH signal was incorrectly removed, but this was mainly due to low SNR of the PA signals, causing incorrect thresholding. Better segmentation methods would be able to improve this.

The gCNR of the PA data could not be assessed. This is due to the fact that PA signals have the highest signal intensity at the center of the source and a decreasing signal intensity toward the edges. Therefore, the PA signal intensity toward the edge of the PA source cannot be distinguished from background noise, which makes it impossible to delineate a region of interest that includes the signal without any background.

For the phantom datasets, the PA signals from the arterioles are at times higher than the PA signals of the aged blood. The reason for the lower PA signals of the IPH channels that contain aged blood is the fact that these receive less optical energy. The factors that may cause this are the IPH channels not being in line with the optical beam or them being partially obstructed by the other channels, which absorb a lot of the optical energy, thereby decreasing the local optical fluence to the IPH channels.

It should be noted that although sealed and aged blood may not have the same photoacoustic spectrum as IPH, this is not a concern as long as the IPH or aged blood have a sufficient PA response at the selected wavelength, as this research is focused on detecting the presence of IPH using a single laser wavelength.

The framework can require some slight tuning of the user-adjustable parameters, but only if the acquisition scheme or application is changed. In the *in vivo* dataset the block-size and moving average window size for the singular value curves were larger than for the *ex vivo* datasets, which was necessary due to the difference in acquisition scheme (no angle compounding, larger field-of-view), as well as the application (probe motion, restricted space in cavity, etc.).

Moreover, due to limitations in the experimental set-up for the *ex vivo* phantom datasets, flow in the arteriole channel was set to a constant 10  mL/h. This made PA signal separation much more challenging than in *in-vivo* conditions for large channel sizes and less challenging for the smallest channel size. Furthermore, the dimensions of the *ex vivo* phantom dataset reduced the maximum block-size that could be tested.

Note that all the results presented in this study were based on a prototype of the integrated US/PA imaging system. Due to a limited number of US elements and limited system memory in the prototype scanner, both number of frames and image quality were limited. Higher imaging quality and a higher number of frames would improve the performance of the filter.

Related to this is the minimum detection size of blood flow, which, as mentioned before, is limited to about 250  μm due to the current system resolution. An improved system with a higher resolution would allow the detection of the small vaso vasorum around the plaque lesions due to neovascularization, and the proposed framework can potentially enable the detection of vulnerable plaques at an early stage, or tracking the progression of plaque development.

Our SVD-based framework is modified further from previous work by Song et al.^30^ to allow processing of interleaved US/PA scans, with a low frame rate of about 10 Hz. The automatic thresholding uses a ratio-based approach for the low-order singular value threshold and the minimum slope for the high-order singular value threshold, which allows the framework to operate at low frame rates, by eliminating the need to use Doppler based thresholds. Furthermore, the smoothing applied to the singular value curves using a moving average filter improves the robustness of the framework proposed. This significantly reduces noise in the curves and reduces discontinuities between adjacent blocks. Any remaining thresholding discontinuities between blocks that are not due to tissue boundaries are removed by the median filter that is applied after thresholding.

Another important factor that influences the performance of the filter is the block-size: a larger block-size improves both execution speed and the IPH detection, at the cost of higher demands on system memory and a slightly lower improvement in image quality. The proposed SVD framework is not limited to square filters which allows for a significant reduction in execution time, by setting the lateral block-size equal to the lateral field-of-view. This decreases execution time from $O(b^{\left(m-p+1)(n-q+1\right)})$ to $O(b^{m-p+1})$ for a m by n matrix, a p by q blocksize, and with b the execution time for a single block.

## 5 Future Work

Due to the limited time of the *in vivo* study, which was caused in part by the COVID-19 pandemic, only a limited number of patient datasets were available. The number was enough for a proof of principle, but a future study could include more patients, providing more biological variance.

In addition, besides the improvement of image quality and IPH detection capability as proposed in this paper, there are more potential applications. One example of such an application would be to monitor uptake of contrast agent in the target tissue.

## 6 Conclusion

The SVD framework can significantly improve US and PA image quality, as demonstrated in an *ex vivo* and *in vivo* setting. Furthermore, when PA signals from hemorrhages were detectable, the framework could identify IPH using a single wavelength in the presence of blood flow and significant wall motion. It can open up new opportunities to detect vulnerable plaques using more economical and cost-effective laser sources and may aid in the clinical translation of PA imaging of vulnerable plaques.

## Code, Data, and Materials Availability

Data and code are not publicly available at this time, but may be obtained from the others upon reasonable request.
